# Single-Molecule Study Reveals How Receptor and Ras Synergistically Activate PI3K*α* and PIP_3_ Signaling

**DOI:** 10.1016/j.bpj.2017.09.018

**Published:** 2017-12-05

**Authors:** Thomas C. Buckles, Brian P. Ziemba, Glenn R. Masson, Roger L. Williams, Joseph J. Falke

**Affiliations:** 1 Molecular Biophysics Program and Department of Chemistry and Biochemistry, University of Colorado, Boulder, Colorado; 2 Medical Research Council, Laboratory of Molecular Biology, Cambridge, United Kingdom

## Abstract

Cellular pathways controlling chemotaxis, growth, survival, and oncogenesis are activated by receptor tyrosine kinases and small G-proteins of the Ras superfamily that stimulate specific isoforms of phosphatidylinositol-3-kinase (PI3K). These PI3K lipid kinases phosphorylate the constitutive lipid phosphatidylinositol-4,5-bisphosphate (PIP_2_) to produce the signaling lipid phosphatidylinositol-3,4,5-trisphosphate (PIP_3_). Progress has been made in understanding direct, moderate PI3K activation by receptors. In contrast, the mechanism by which receptors and Ras synergistically activate PI3K to much higher levels remains unclear, and two competing models have been proposed: membrane recruitment versus activation of the membrane-bound enzyme. To resolve this central mechanistic question, this study employs single-molecule imaging to investigate PI3K activation in a six-component pathway reconstituted on a supported lipid bilayer. The findings reveal that simultaneous activation by a receptor activation loop (from platelet-derived growth factor receptor, a receptor tyrosine kinase) and H-Ras generates strong, synergistic activation of PI3K*α*, yielding a large increase in net kinase activity via the membrane recruitment mechanism. Synergy requires receptor phospho-Tyr and two anionic lipids (phosphatidylserine and PIP_2_) to make PI3K*α* competent for bilayer docking, as well as for subsequent binding and phosphorylation of substrate PIP_2_ to generate product PIP_3_. Synergy also requires recruitment to membrane-bound H-Ras, which greatly speeds the formation of a stable, membrane-bound PI3K*α* complex, modestly slows its off rate, and dramatically increases its equilibrium surface density. Surprisingly, H-Ras binding significantly inhibits the specific kinase activity of the membrane-bound PI3K*α* molecule, but this minor enzyme inhibition is overwhelmed by the marked enhancement of membrane recruitment. The findings have direct impacts for the fields of chemotaxis, innate immunity, inflammation, carcinogenesis, and drug design.

## Introduction

Receptor-Ras-PI3K-PIP_3_ signaling is central to an array of essential pathways. Localized PIP_3_ signals are generated at the leading-edge membrane of chemotaxing cells, including leukocytes migrating toward a site of infection or inflammation (1). PIP_3_ signals also play central roles in cell growth and survival pathways (2). Many, perhaps most, human cancers are linked to excessive PIP_3_ production arising from oncogenic mutations in receptor, Ras, or PI3K components (2, 3, 4, 5, 6).

**Figure 1:**
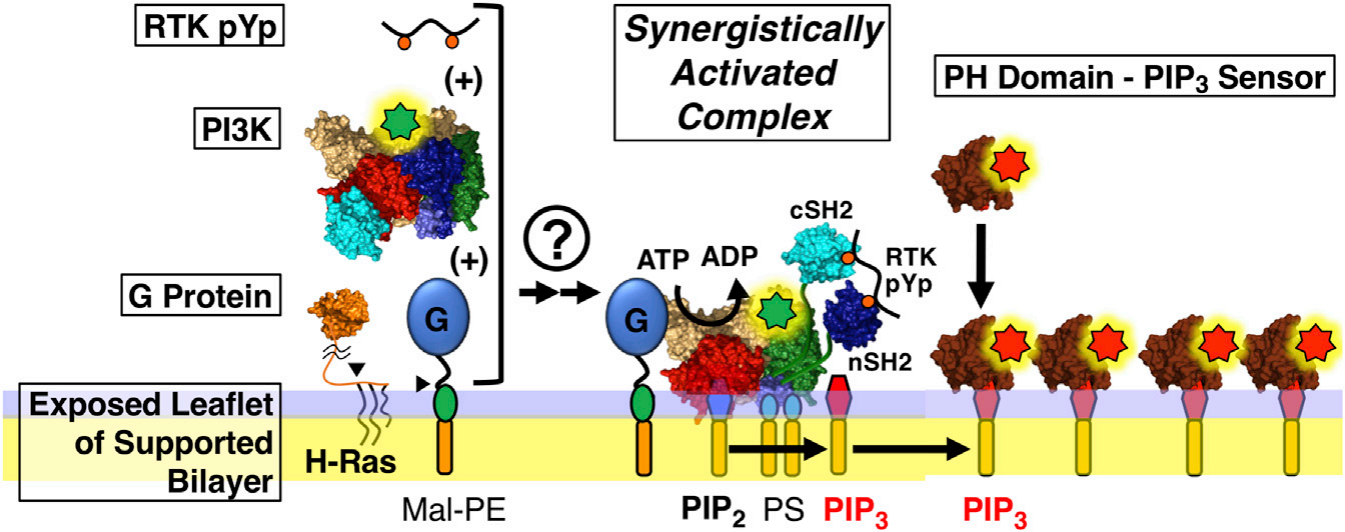
Molecular schematic of the PI3K complex synergistically activated by receptor and G-protein in the single-molecule kinase activity assay. Shown is a schematic depiction of the fully active PI3K*α* complex assembled on the supported lipid bilayer. In this complex, PI3K*α* is synergistically activated by both 1) an RTK-derived phospho-Tyr peptide (RTK-pYp) based on the activation loop of the platelet-derived growth factor receptor and phosphorylated at specific Tyr residues, and 2) a small G-protein (H-Ras) anchored to maleimide lipid. The arrowhead indicates native Cys 181, which is a palmitoylation site in wild-type H-Ras and is sufficient for functional, covalent coupling to maleimide lipid in the in vitro single-molecule kinase assay. Single-molecule assays monitor stable docking of fluorescent PI3K*α* molecules (*green star*) to the membrane, and PI3K*α*-catalyzed production of the product PIP_3_ signaling lipid. Each product PIP_3_ molecule is detected by the binding of fluorescent GRP PH domain (*red star*) added in excess as a PIP_3_ sensor. The assay monitors the initial rate of PIP_3_ production when <2% of the substrate PIP_2_ population is converted to PIP_3_. At these low levels of PIP_3_, product rebinding to PI3K*α* is negligible; thus, sequestration of the product PIP_3_ by the sensor protein is non-perturbing. To see this figure in color, go online.

Previous mechanistic studies have shown that receptor tyrosine kinase (RTK) activation of the dominant class IA PI3K family modulates the interactions between the two subunits of the PI3K heterodimer (5, 7, 8, 9, 10). This modulation occurs when receptor phospho-Tyr residues, located in a flexible cytoplasmic loop, bind to one or both inhibitory SH2 domains of the p85 regulatory subunit (Fig. 1). The resulting phospho-Tyr binding displaces the SH2s from the p110 catalytic subunit, triggering a conformational change that exposes lipid binding surfaces and activates the kinase domain, thereby generating modest levels of membrane binding and kinase activity.

In cells and in vitro, receptors and G-proteins have been observed to act in concert to synergistically stimulate PI3Ks and PIP_3_ production (11, 12, 13). In cells, some stimuli may simultaneously activate multiple, parallel G-protein responses that stimulate multiple PI3K populations to produce additive PIP_3_ signals, making it difficult to ascertain whether synergy requires direct, simultaneous binding of receptor and G-protein to PI3K (12, 13). In vitro, it has recently been demonstrated that the simultaneous presence of receptor phospho-Tyr and G-protein does indeed generate synergistic activation of class IA PI3Ks (11), consistent with the simultaneous binding of both ligands to PI3K at their distinct binding sites previously identified by crystallographic studies (6, 10, 14).

Two competing models have been proposed for the molecular mechanism by which G-protein amplifies the modest PI3K activation triggered by receptor alone to generate dramatic, synergistic activation. In the membrane recruitment model, G-protein enhances PI3K binding to the membrane, yielding increased membrane density of the active lipid kinase (15, 16). In the enzyme activation model, G-protein increases the specific activity (or turnover number) of each membrane-bound, G-protein-associated, kinase molecule (14, 17). The two mechanisms are not mutually exclusive, so one could dominate or both could contribute. Current evidence is inconclusive. For example, Ras isoforms do recruit PI3K to the membrane in cells (15). On the other hand, Ras binding does trigger PI3K conformational changes detected in crystallographic and hydrogen-deuterium exchange studies that could, in principle, modulate its enzyme activity (11, 14). Previous studies take opposite sides or conclude that the issue is an open question (11, 13, 14, 16). To date, the two models have not been resolved for any G-protein-PI3K regulatory pair.

To address these mechanistic questions, this study employs single-molecule total internal reflection fluorescence microscopy (TIRFM) to investigate a representative receptor-Ras-PI3K-PIP_3_ signaling module reconstituted from four protein/peptide components and two lipid components on a supported lipid bilayer: 1) A widely employed soluble phospho-Tyr peptide (pYp) is used to mimic the flexible, regulatory phospho-Tyr loop of an RTK receptor (7, 8, 9, 10, 18, 19). This peptide possesses phospho-Tyr at one or both conserved phosphorylation sites and is derived from the platelet-derived growth factor receptor, an RTK central to chemotaxis and inflammation. 2) The membrane-anchored, small G-protein H-Ras is employed as a representative Ras isoform. H-Ras is central to leukocyte transmigration and is linked to at least 65 oncogenic mutations (http://cancer.sanger.ac.uk/cosmic; https://www.cancer.gov/research/key-initiatives/ras/about) (20, 21). More broadly, mutations in this and other Ras isoforms are linked to >25% of human cancers (http://cancer.sanger.ac.uk/cosmic; https://www.cancer.gov/research/key-initiatives/ras/about) (17). 3) PI3K*α* is the most prevalent and oncogenic PI3K isoform (6, 22). This heterodimeric lipid kinase is composed of catalytic (p110*α*) and regulatory (p85*α*) subunits, and is activated by both RTKs and Ras isoforms (6, 7). PI3K*α* is involved in leukocyte chemotaxis and inflammation (1, 23) and is linked to 255 oncogenic mutations (http://cancer.sanger.ac.uk/cosmic; https://www.cancer.gov/research/key-initiatives/ras/about) (3, 15, 22, 24). 4–6) The anionic lipids phosphatidylserine (PS) and PIP_2_ serve as PI3K*α* lipid binding targets; the latter is also the substrate for PIP_3_ production (7, 25). 7) General receptor for phosphoinositide (GRP1) pleckstrin homology (PH) domain is a high-affinity PIP_3_ sensor employed to specifically detect product PIP_3_ even in the presence of high PIP_2_ densities (26).

The findings reveal strong, direct synergy between simultaneous receptor phospho-Tyr and H-Ras activators. As previously observed, receptor phospho-Tyr residues are required for modest activation of PI3K*α* lipid binding and PIP_3_ production (9, 10). When membrane-bound H-Ras is also present, the resulting receptor-G-protein synergy drives much stronger PI3K*α* activation, and the findings show that this synergy is generated by the membrane recruitment mechanism. The enzyme activation mechanism does not contribute to this activation; instead, the findings reveal that H-Ras binding significantly inhibits the specific enzyme activity of membrane-bound PI3K*α*.

## Methods

### Reagents

Except where noted otherwise, fluors, synthetic lipids, synthetic phospho-Tyr peptide containing the activation loop sequence of platelet-derived growth factor receptor (an RTK), and other reagents and materials were obtained as previously described from the same suppliers (18, 19). In addition, the maleimide headgroup phospholipid dioleolyl-phosphoenthanolamine-N-[4-(p-maleimidomethyl)cyclohexane-carboxamide] (DOPE-MCC or Mal-PE) was obtained from Avanti Polar Lipids (Alabaster, AL). Functional PI3K*α* and GRP1 PH domain were obtained and labeled with fluorophore by a gentle, enzymatic procedure at their Sfp labeling tag, as previously described (18, 19).

### Preparation of supported lipid bilayers possessing membrane-anchored H-Ras

A published method was employed to express and purify unlipidated H-Ras from *Escherichia coli* (27) (Fig. S1). After confirmation of identity by mass spectroscopy analysis, the desired nucleotide (generally non-hydrolyzable GTP analog GMPPNP, or, where indicated, GTP or GDP) was loaded into H-Ras by nucleotide exchange using a standard, high-EDTA exchange method (27). The bilayer lipid mixture employed was DOPE/DOPS/DOPIP_2_/DOPE-MCC in a mole ratio of 73:25:1:1. Our previously described procedures (18, 19, 26, 28, 29) were used to deposit a homogeneous bilayer composed of these lipids on ultra-clean glass, yielding a supported lipid bilayer (28). The purified, nucleotide-loaded H-Ras was covalently coupled to the headgroups of DOPE-MCC via its native membrane-anchoring Cys residues 181 and 184 or, where noted, via only Cys 181 (see Table S1). After washing the bilayer to remove all remaining free H-Ras, the surface density and diffusion speed of anchored H-Ras were measured by single-molecule TIRFM using a fluor-tagged, antibody to detect and count each membrane-bound H-Ras molecule (see Fig. S2; Table S1). Functional analysis revealed that the membrane-anchored H-Ras is active in PI3K*α* recruitment and kinase assays and exhibits the expected nucleotide specificity for activation by GTP or GTP analog).

### Single-molecule TIRFM measurements

TIRFM experiments were carried out at 21.5 ± 0.5°C on an objective-based TIRFM instrument, as described previously (26, 28). The instrument utilized a Nikon (Melville, NY) TE2000U inverted TIRF microscope; a Nikon Apochromat 60×, NA 1.49 TIRF oil immersion objective; and a CNI-Laser 300 mW, 532 nm, diode-pumped solid-state laser model MGLIII-532-300 mW. The laser power exiting the objective was reduced by pre-microscope neutral density filters to 2.3 mW for observation (power measured in epi mode before switching to TIRFM). Sample fluorescence emerging from the 600 nm shortpass emission filter was captured by an Evolve electron-multiplying charge-coupled device camera (Photometrics, Tucson, AZ).

TIRFM supported bilayers were first washed with TIRF assay buffer (100 mM KCl, 20 mM HEPES (pH 6.9), 15 mM NaCl, 5 mM glutathione, 2.0 mM EGTA, 1.9 mM Ca^2+^, and 0.5 mM Mg^2+^, where this Ca^2+^/Mg^2+^ buffering system yields 10 *μ*M free Ca^2+^ and 0.5 mM free Mg^2+^), then imaged before and after adding a concentrated mixture of bovine serum albumin (BSA) and 3-[(3-cholamidopropyl)dimethylammonio]-1-propanesulfonate (CHAPS) to final concentrations of 100 *μ*g/mL and 0.05%, respectively. After this addition, only a few dim, rapidly dissociating fluorescent contaminants were typically observed on the bilayer before protein addition, and they were easily eliminated from the data, as described below. Occasionally, the contaminant level was excessive and the reagents (starting with the lipids) were remade.

After confirmation of minimal contamination, stabilizers, proteins, and nucleotides were added to the bilayer as needed and were equilibrated for 3 min. These added components included a low level of BSA (100 *μ*g mL^−1^ BSA final concentration) to block sticky surfaces that could absorb the dilute proteins (30). Other additions were included as appropriate, including CHAPS (0.05%) to prevent PI3K aggregation, phospho-Tyr peptide (saturating) for PI3K activation, guanosine nucleotide (1 mM) for maintaining H-Ras in its nucleotide-loaded state (in experiments employing this protein), ATP (1 mM) as a PI3K substrate for phosphorylation of PIP_2_, and GRP PH domain (800 pM) as a sensor for PIP_3_ detection. Where needed, aliquots of PI3K were thawed on ice and diluted into stabilizing buffer (125 mM NaCl, 20 mM HEPES (pH 7.2), 25% glycerol, 4 mM TCEP, 0.05% CHAPS, and 100 *μ*g mL^−1^ BSA) that maximizes their stability on ice until they are diluted, just before use, to the final concentration (2 nM) in the buffer above the membrane.

To minimize contributions from small numbers of immobile unfolded proteins bound to a low density of membrane defects, a bleach pulse of ∼5-fold higher power than that used for imaging was applied for ∼15 s; then fluorescence was allowed to return to a steady state for at least 60 s before data acquisition. For each sample, a set of two to four movie streams were acquired at a frame rate of 20 frames/s and a spatial resolution of 4.2 pixels/*μ*m on the home-built instrument, using NIS Elements Basic Research (Nikon).

### Single-molecule diffusion tracking

As in our previous studies (26, 28, 31), diffusion trajectories of single-protein molecules were tracked and quantitated using the Particle Tracker plugin for ImageJ (32), yielding a per-frame quantitation of particle position and brightness. The resulting data were then imported into Mathematica for further analysis. Only particles possessing fluorescence intensities within a defined range were included in the analysis, thereby eliminating bright fluorescent contaminants/protein aggregates and dim, non-protein contaminants. Additional displacement-based exclusions removed immobile particles, rapidly dissociating particles, and overlapping tracks for which particle identity is lost. All exclusions were described and validated previously (26, 28, 31).

### PI3K membrane binding densities

To investigate the mechanisms of PI3K*α* activation by receptor-derived pYp and/or membrane-anchored H-Ras, we employed our recently published single-molecule methods to quantify the effects of activators on PI3K*α* surface density and lipid kinase activity (18, 19). Binding measurements focused on stable PI3K*α* complexes bound to the membrane for at least five consecutive 20 ms movie frames, yielding tracks lasting ≥100 ms.

To quantify the average density of PI3K*α* on the membrane surface in a given TIRF movie, the number of single-particle tracks (defined as described above) in a given field of view was determined for each movie frame, then averaged over all frames. Bleaching of individual tracks or the bulk population was not a major issue, since the average residence time on the membrane before dissociation was short compared to the average bleach time; as a result, fluorescent proteins dissociate from the membrane before bleaching and are replaced from the bulk population, which lies predominantly outside the TIRF excitation field. Under our experimental conditions, the average time to bleach of the Alexa 555 fluor was ≥25 s (26, 28), which is at least 10-fold longer than the average bound-state lifetime of any labeled protein in this study.

### PI3K kinase activities

A single-molecule kinase assay was employed to quantify PI3K*α* lipid kinase activity and PIP_3_ production, as previously described (19). The method counts all single molecules of product PIP_3_ generated by the PI3K lipid kinase reaction, using a saturating concentration of fluor-tagged, GRP-PH domain (800 pM) to bind and detect each PIP_3_ molecule generated on the membrane surface. After PI3K was added to the chamber (see Single-Molecule TIRFM Measurements above), ATP (1 mM) was added from a buffered stock (assay buffer containing 100 mM ATP and 82.5 mM Mg^2+^) to start the kinase reaction. Subsequently, fluor-tagged PH domain tracks were quantified as previously described at five time points (18, 19). To determine the PI3K*α* specific activity, the resulting net rate of PIP_3_ production is divided by the average density of PI3K*α* determined by the above binding assay (with appropriate correction for the PI3K fluorescence labeling efficiency).

### Statistics

PI3K*α* is a large, 208 kDa heterodimeric enzyme with nine structural domains and retains function well when stored in frozen, single thaw aliquots. However, thawed aliquots exhibit variability in activity that represent the major limitation on precision in studies of membrane binding, kinase activity, and specific activity. Thus, large numbers of replicates were carried out on multiple days to allow rigorous statistical analysis. Specifically, for each measured parameter, *n* means were determined, where each mean averaged 3+ replicates carried out on the same day. Error bars represent the standard error of these *n* means determined on 5–15 different days (*n* = 5–15).

## Results

### Mimicking the cellular signaling pathway, and production of membrane-anchored H-Ras

The single-molecule approach enables use of near-physiological protein concentrations and target lipid densities designed to approximate those found in the cellular pathway. Thus, this study employed a PI3K*α* concentration (2 nM) of the same order as its physiological concentration (estimated 3 nM (33)), together with a pYp level (saturating) to simulate PI3K*α* bound to the regulatory loop of its phospho-activated RTK receptor. The H-Ras coupling protocol yields a surface density of monomeric H-Ras (1 per *μ*M^2^; see Fig. S2; Table S1) ∼30-fold lower than the surface density reported in cells (34, 35). However, the in vitro system presented here fully loads membrane-anchored H-Ras with GTP or non-hydrolyzable GTP analog, yielding a surface density of active, monomeric H-Ras similar to that expected for moderate H-Ras signals in cells where only a fraction of the membrane-anchored population is activated by GTP loading (36). Moreover, the specific kinase activity (turnover number) of PI3K*α* bound to pYp and H-Ras on the membrane surface is the same, within error, at different H-Ras surface densities (Fig. S4). These findings are consistent with a simple picture in which, as the H-Ras surface density increases, the density of membrane-bound PI3K*α* and its net kinase activity increase linearly, whereas the specific activity of membrane-bound PI3K*α* remains unchanged because its activation mechanism is density independent. The fluorescent GRP1 PH domain employed to detect PIP_3_ molecules generated by PI3K*α* is present in the in vitro single-molecule kinase assays at levels (800 pM) sufficient to fully bind all product PIP_3_ molecules, just as sufficient PH domain proteins are present in cells to bind all product PIP_3_ (37). Finally, the supported bilayer densities of the anionic background lipid PS (mole fraction 25%) and the target-substrate lipid PIP_2_ (mole fraction 1%) are similar to those estimated for the plasma membrane cytoplasmic leaflet (38, 39).

Our previous single-molecule TIRFM studies have characterized the membrane binding and two-dimensional diffusion of the PI3K*α* and GRP PH domain proteins on supported bilayers, revealing that PI3K*α* possesses extensive bilayer contacts involving multiple lipids, whereas GRP PH domain binds a single PIP_3_ and diffuses like a single lipid, since the lipid drag against the bilayer largely controls the diffusion speed. This study carries out the first single-molecule analysis, to our knowledge, of PI3K regulation by H-Ras, employing a recently developed approach (27) to covalently anchor functional H-Ras to the supported lipid bilayer. The membrane coupling procedure targeted two native Cys residues that are located near the C-terminus of the hyper-variable region (HVR) (Fig. S1) and are palmitoylated in native H-Ras, where they serve as plasma membrane anchors. Here, both of these Cys residues were employed as supported bilayer anchors by covalently coupling them to a maleimidyl-modified phospholipid headgroup.

Single-molecule TIRFM was used to characterize the density and diffusion speed of the maleimide-lipid-anchored H-Ras on the supported bilayer surface (Fig. S2; Table S1). The measured density was ∼1 H-Ras/*μ*m^2^ (Table S1). Analysis of the two-dimensional diffusion tracks revealed two subpopulations of membrane-anchored monomeric H-Ras with fast and slow diffusion speeds, corresponding to the frictional drag of one and two coupled lipids (26, 40), respectively (Table S1). The latter H-Ras subpopulation coupled to two lipids via C181 and C184 was found to be the larger, predominant subpopulation (Table S1). The resulting membrane-anchored H-Ras retains the native 14 residue, unstructured tether between membrane-anchored C181 and the folded GTPase domain (Figs. 1 and S1).

### Synergistic activation of PI3K*α* by receptor-derived phospho-Tyr peptide and H-Ras

**Figure 2:**
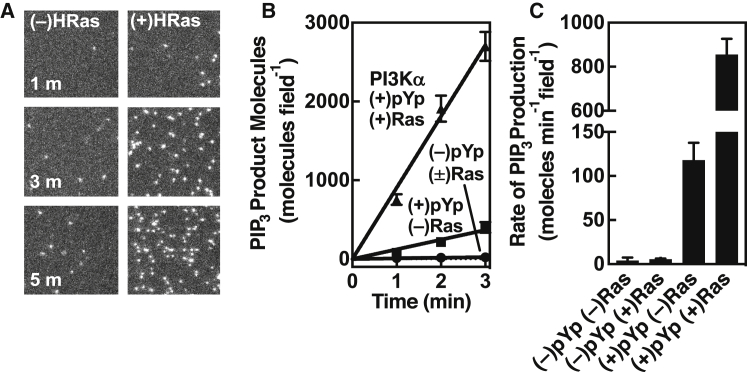
Effect of receptor-derived pYp and H-Ras on net PI3K*α* kinase activity and PIP_3_ production. (*A*) TIRFM images of pYp-activated PI3K*α* lipid kinase activity on a target bilayer surface. Shown is the accumulation of product PIP_3_ lipid detected by the fluor-labeled PIP_3_ sensor (GRP PH domain), with and without membrane-anchored H-Ras. Each image is a square section (121 pixels or 29.0 *μ*m per side) of the monitored TIRFM area (256 pixels or 61.4 *μ*m per side). (*B*) Time course plotting the increasing number of PIP_3_ molecules per field under four activating conditions, illustrating strong synergy between the pYp peptide and H-Ras in activating PI3K*α* kinase and PIP_3_ production. (*C*) The average slopes of these time courses, yielding average net rates of PIP_3_ production. Each average rate was generated from three replicates on each of nine or more different days (*n* = 27–36). In all cases in (*A*)–(*C*), H-Ras is loaded with non-hydrolyzable GTP analog GMPPNP.

To probe the activation of PI3K*α* lipid kinase, we employed our previously described single-molecule TIRFM assay of lipid kinase activity (18, 19). This assay directly monitors the number of individual product PIP_3_ molecules produced as a function of time by catalytically active, membrane-bound PI3K*α* (Figs. 1 and 2).

Fig. 2 reveals strong, direct synergy in PI3K*α* activation between the receptor-mimicking pYp and membrane-bound H-Ras loaded with non-hydrolyzable GTP analog. Notably, in the absence of pYp, H-Ras alone yields little or no activation of PI3K*α*-catalyzed PIP_3_ production. In contrast, saturating pYp alone triggers an ∼30-fold increase (*p* < 0.002) in the net rate of PIP_3_ production, indicating moderate PI3K*α* activation in the absence of H-Ras. Together, however, pYp and H-Ras synergistically speed net PIP_3_ production by nearly an order of magnitude (8 ± twofold) (*p* < 0.001) more than pYp alone, or ∼200-fold (*p* < 0.001) more than H-Ras alone. Controls show that the additional PI3K*α* activation triggered by H-Ras in the presence of pYp requires membrane-anchored H-Ras, is GTP-regulated, and is specific (Fig. S3). Additional controls show that the pYp and H-Ras activators have no effect on the binding of the PIP_3_ sensor GRP1 PH domain to PIP_3_ on the supported bilayer surface (Fig. S5), confirming that the assay accurately measures the effects of these activators on PI3K*α* lipid kinase activity.

### Synergy dramatically increases the membrane density of stably bound PI3K*α* molecules

To investigate the unknown mechanism by which H-Ras dramatically amplifies the moderate PI3K*α* activation observed for pYp alone, we measured the effect of both activators on fluor-labeled PI3K*α* membrane binding and specific kinase activity.

**Figure 3:**
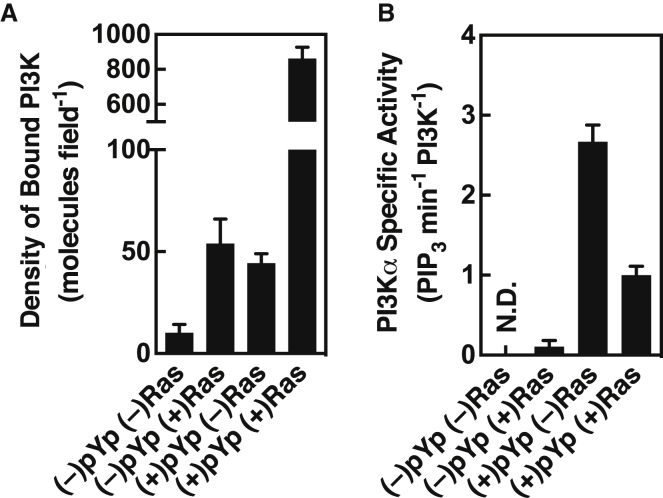
Effect of pYp and H-Ras on PI3K*α* membrane recruitment and on the specific kinase activity of the membrane-bound enzyme. (*A*) Surface density of fluor-labeled PI3K*α* measured by single-molecule TIRFM under the indicated activation conditions. Each density was an average over three replicates on each of five different days (*n* = 15). (*B*) Specific activity of membrane-bound PI3K*α* under each activating condition, calculated as the ratio of net PIP_3_ production (Fig. 2*C*) to the kinase surface density (*A*). This specific activity ratio could not be determined for the (–)pYp (–)Ras condition due to the low density of kinase on the bilayer surface. In all cases, in both (*A*) and (*B*), H-Ras is loaded with non-hydrolyzable GTP analog GMPPNP.

Fig. 3 *A* shows that either pYp or H-Ras alone triggers moderate densities of long-lived PI3K*α* molecules on the membrane surface, whereas the two activators together exhibit strong synergy and dramatically increase the surface density of kinetically stable PI3K*α* complexes. Measurements focused on stable PI3K*α* diffusion tracks lasting ≥100 ms. Addition of saturating pYp increases the density of PI3K*α* 4 ± twofold (*p* < 0.001) on supported bilayers lacking H-Ras, comparable to our recently published result on membranes of nearly identical lipid composition (19). Similarly, in the absence of pYp, supported bilayers possessing membrane-anchored H-Ras yielded 5 ± twofold (*p* < 0.001) higher PI3K*α* surface density than bilayers lacking H-Ras. Strikingly, when both pYp and membrane-anchored H-Ras were present, the PI3K*α* surface density was 80 ± 30-fold (*p* < 0.001) greater than in the absence of both activators, or 20 ± 2.5-fold (*p* < 0.001) greater than pYp alone.

**Figure 4:**
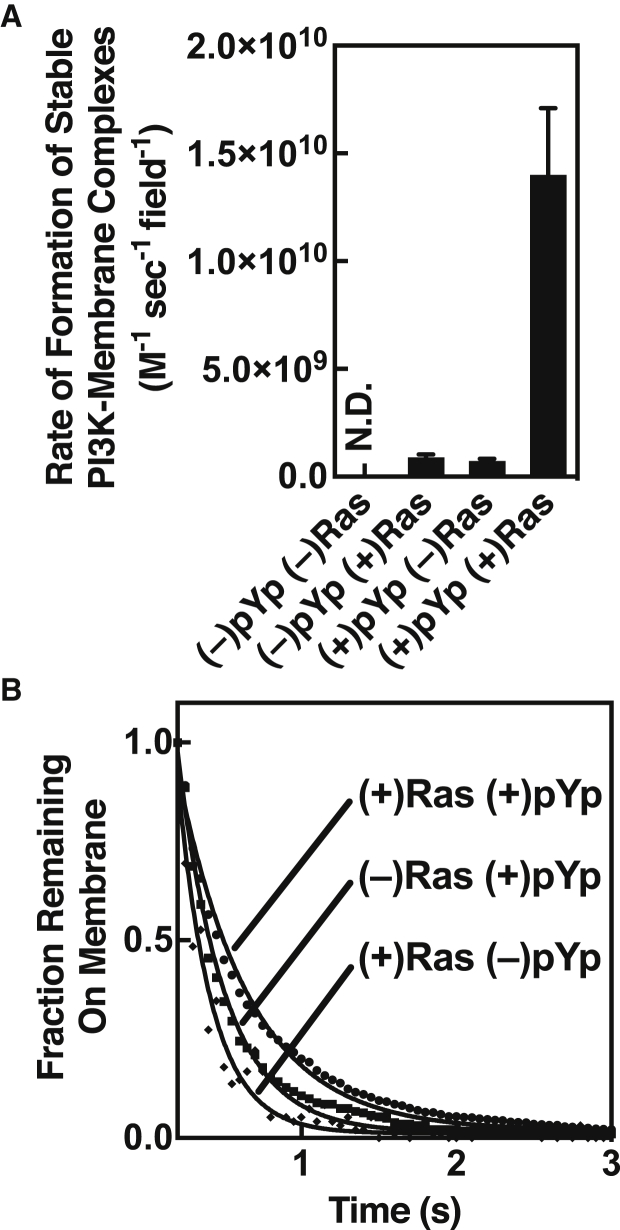
Effect of pYp and H-Ras on PI3K*α* association-dissociation kinetics. (*A*) Relative appearance rates of stable, fluor-PI3K*α* single-particle diffusion tracks were measured under different activating conditions. These appearance rates were used to calculate the formation rate of active, stably bound PI3K*α* complexes on the membrane surface as follows. The number of PI3K*α* tracks with bound-state lifetimes exceeding 100 ms in a TIRFM movie was determined, then divided by movie time and bulk PI3K*α* concentration to yield an operationally defined appearance rate. Each rate was generated from groups of three replicate movies acquired on each of five different days (*n* = 15). (*B*) Off rates of fluor-PI3K*α* from the membrane surface, calculated by binning the bound-state lifetimes of stable PI3K*α* single-particle diffusion tracks at least 150 ms in length and fitting with a single-exponential decay. Each decay curve was generated from groups of three replicate movies acquired on each of five different days (*n* = 15). The resulting exponential decay constants were: 2.9 ± 0.6 s^−1^ for (–)pYp (+)Ras, 2.5 ± 0.6 s^−1^ for (+)pYp (–)Ras, and 1.9 ± 0.2 s^−1^ for (+)pYp (+)Ras. No kinetic data could be obtained for (–)pYp (–)Ras due to its very low density on the bilayer surface. In all cases, in both (*A*) and (*B*), H-Ras is loaded with non-hydrolyzable GTP analog GMPPNP.

The strong synergy observed between pYp and H-Ras in driving increased equilibrium levels of PI3K*α* on the membrane surface arises both from faster formation of stable PI3K*α* membrane complexes and slower dissociation. Fig. 4 *A* shows that the apparent formation rates of stable PI3K*α* single-particle tracks with bound-state lifetimes exceeding 100 ms are equally slow, within error, for either activator alone. By contrast, relative to pYp alone, combined peptide and H-Ras greatly increases the formation rate of stable tracks 19 ± fivefold (*p* < 0.008) and reproducibly decreases their dissociation rate by a small but significant factor (1.4 ± 0.3-fold; *p* = 0.003). Together, these kinetic effects fully account for the 20 ± 2.5-fold (*p* < 0.001) greater density of membrane-bound PI3K*α* observed when pYp and H-Ras act synergistically.

### Synergy significantly decreases the specific activity of membrane-bound PI3K*α* molecules

Surprisingly, although pYp and membrane-anchored H-Ras synergize to increase the density of stably bound PI3K*α* molecules on the supported bilayer surface, this synergy also decreases the specific lipid kinase activity of the membrane-bound enzyme. Fig. 3 *B* presents specific lipid kinase activities calculated by determining the ratio of the net rate of PI3K*α*-catalyzed PIP_3_ production to the surface density of PI3K*α* (Figs. 2 *C* and 3 *A*). The specific kinase activity of the average membrane-bound molecule activated by pYp and H-Ras together is, unexpectedly, 2.6 ± 0.6-fold (*p* < 0.001) lower than observed for activation by pYp alone, indicating that the formation of the H-Ras-PI3K complex significantly inhibits the catalytic activity of membrane-bound, pYp-activated PI3K*α*.

Overall, the findings reveal that the 8 ± twofold (*p* < 0.001) faster net PIP_3_ production observed for synergistic pYp and H-Ras activation, relative to pYp alone, arises from both the 20 ± 2.5-fold (*p* < 0.001) greater PI3K*α* membrane recruitment noted above, combined with the 2.6 ± 0.6-fold (*p* < 0.001) slower specific kinase activity. Notably, H-Ras alone yields moderate levels of PI3K*α* surface density but fails to generate any detectable kinase activation. Thus, both in the absence and presence of H-Ras (10, 12, 18, 19), pYp plays an essential role in making PI3K*α* competent for lipid association and stimulating lipid kinase activity. These results fully support the currently held view that association of receptor phospho-Tyr residues with the inhibitory SH2 domains of the PI3K p85 regulatory domain is required to make the p110 catalytic domain accessible for PIP_2_ binding and catalysis (5, 7, 8, 9, 10). The observation that H-Ras significantly inhibits the specific kinase activity of pYp-activated PI3K*α* emphasizes that the contribution of H-Ras to synergistic G-protein-receptor activation arises purely from a membrane recruitment mechanism, with no contribution from an enzyme activation mechanism.

## Discussion

The findings presented here show a strong, direct synergy between receptor-derived pYp and monomeric, membrane-anchored H-Ras in activating net PIP_3_ production by PI3K*α* lipid kinase. Previously, synergy between receptor and Ras activation of specific PI3K isoforms, including PI3K*α*, has been proposed by studies in cells, where it is difficult to rule out indirect synergies arising from multi-pathway activation (12, 13). A recent in vitro study observed direct, synergistic activation of class IA PI3K kinase activity by simultaneously added pYp and H-Ras (11). Notably, however, this study did not quantify the effect of activators on PI3K membrane density, nor on the specific kinase activity of the membrane-bound enzyme; thus, it was not able to resolve the long-standing controversy between the membrane recruitment and enzyme activation models for the mechanism of synergistic activation.

The findings presented here resolve this long-standing controversy and reveal that H-Ras contributes to synergistic PI3K*α* activation via a membrane-recruitment mechanism, without any detectable contribution from enzyme activation. Relative to pYp alone, H-Ras interactions both speed the formation of kinetically stable, membrane-bound pYp-PI3K*α* complex by a factor of 19 ± fivefold (*p* < 0.008), and slightly but significantly slow the dissociation of PI3K*α* from membrane by 1.4 ± 0.3-fold (*p* = 0.003). At the same time, binding to H-Ras significantly decreases, rather than increases, the specific kinase activity of the membrane-bound pYp-PI3K*α* complex by 2.6 ± 0.6-fold (*p* < 0.001). Thus, the H-Ras contribution to synergistic PI3K*α* activation arises purely from its ability to dramatically increase the surface density of active lipid kinase. Notably, H-Ras alone drives only a modest increase in PI3K*α* surface density and triggers no measurable kinase activation, reiterating the known requirement for receptor phospho-Tyr binding to the PI3K inhibitory SH2 domains to make the enzyme competent for bilayer and PIP_2_ binding, as well as catalytic activity (5, 7, 8, 9, 10, 12, 18, 19).

**Figure 5:**
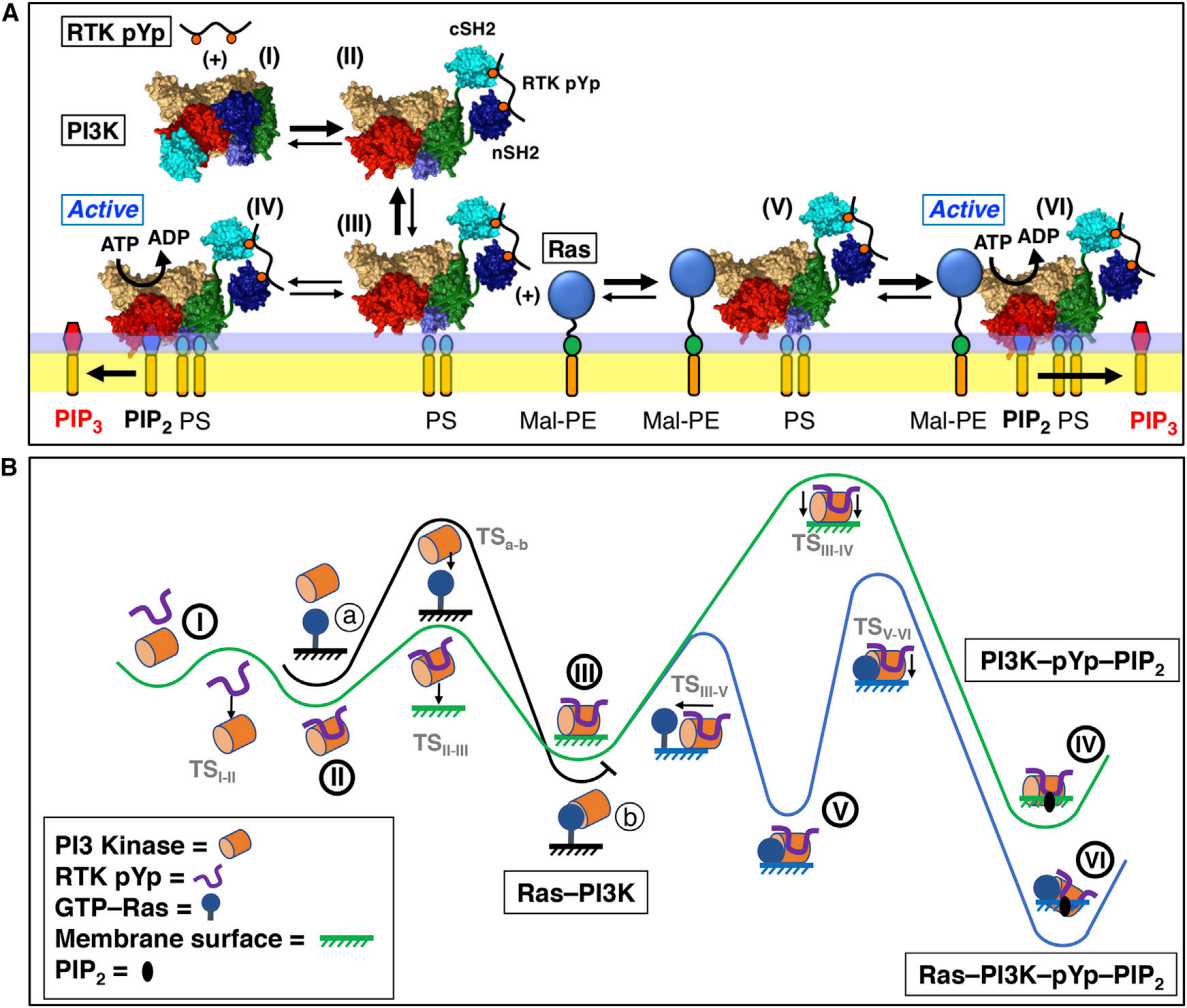
Working model for the mechanism of synergistic PI3K*α* activation by receptor-derived pYp and H-Ras. Shown are two different depictions of the same mechanistic model in which pYp peptide and H-Ras synergistically activate PI3K*α* by enhancing its membrane recruitment. (*A*) Kinetic scheme depicting structural cartoons for each state in the hypothesized multi-step activation reaction, with dominant rates highlighted in bold. Activation begins with the binding of free PI3K*α* in solution (I) to the receptor-derived pYp (RTK pYp), present in excess. One or both inhibitory SH2 domains bind pYp and dissociate from the PI3K*α* catalytic domain, exposing its PIP_2_ binding and catalytic sites (9, 10). The resulting free pYp-activated PI3K*α* complex (II) docks to the membrane, yielding an inactive pYp-PI3K*α* complex transiently bound to the membrane surface in a shallow surface-bound state (III). This transient surface complex (III) rapidly dissociates and returns to solution (II) unless converted to a stable, membrane-bound pYp-PI3K*α* complex by additional membrane interactions, such as deeper penetration into the bilayer (IV). The resulting stable, membrane-bound pYp-PI3K*α* complex (IV) is fully active and displays the maximum specific kinase activity. Alternatively, if present, membrane-anchored H-Ras can rapidly bind to the transient surface complex (III) and catalyze its conversion to the stably bound, deeply penetrating, active Ras-pYp-PI3K*α* complex (V). Such catalytic assistance explains the faster rate of formation of the Ras-bound stable, active complex (V) compared to Rasless stable, active complex (IV). Notably, the Ras-bound complex (V) exhibits two unexpected properties, leading to the hypothesis that Ras binding perturbs the optimal membrane docking geometry displayed by pYp-PI3K*α* in the Rasless complex (IV). First, the rate of Ras-bound complex (V) dissociation from membrane is only slightly slower than that of Rasless complex (IV), indicating that the former complex does not possess the full kinetic stability expected for simple additivity of the binding free energies for pYp-PI3K*α* binding to H-Ras plus pYp-PI3K*α* binding to membrane. Second, the specific kinase activity of the Ras-bound complex (VI) is, surprisingly, significantly lower than that of the Rasless complex (IV). These unexpected features are both consistent with a Ras-bound state (VI) in which structural constraints prevent the optimal interactions of pYp-PI3K*α* with Ras and the membrane simultaneously (see text). Further single-molecule studies will test this working model and determine the rate constants of individual steps. (*B*) Reaction coordinate depiction of the kinetic scheme shown in (*A*), using the same numbering scheme for intermediates (I) through (VI). Also shown is the unproductive path a-to-b for the absence of pYp, wherein PI3K*α* is able to bind weakly to Ras but the lack of phospho-Tyr binding prevents release of the inhibitory SH2 domains bound to the catalytic domain. As a result, the SH2 domains continue to block PI3K membrane binding and kinase activation. To see this figure in color, go online.

Fig. 5, *A* and *B*, presents the simplest kinetic scheme consistent with these data, and the corresponding reaction-coordinate free-energy profile, able to explain the observed kinetic and thermodynamic contrasts between PI3K*α* activation by the two activators, alone and in synergy. In the presence of receptor-derived phospho-Tyr peptide, the pYp first binds to the SH2 domains of the free kinase (I), triggering a conformational change that displaces the inhibitory SH2 domains from the catalytic subunit, thereby activating the lipid binding surfaces of PI3K*α* (5, 7, 8, 9, 10, 12, 18, 19) to yield the docking-competent free kinase (II) (Fig. 5 *A*). It has long been established that this specific pYp binding to the SH2 domains requires both sequence motifs and phospho-Tyr residues on the receptor-derived peptide (41, 42). The resulting pYp-PI3K*α* complex is then hypothesized to bind via an electrostatic mechanism to the anionic membrane surface, where negative charge is provided mainly by PS, yielding a transient, weakly bound surface state (III). This transient state (III) is hypothesized to undergo two-dimensional diffusion (analogous to the electrostatic surface search of PH domains for PIP_3_ (27)) on the membrane surface. Usually it dissociates, but sometimes it binds PIP_2_ and penetrates more deeply into the bilayer to yield a stably bound, kinase-active state (IV). In the presence of H-Ras, the pYp-PI3K*α* complex can encounter and bind H-Ras, either via its free state in solution (II) or via its transient surface state (III). The resulting binding of pYp-PI3K*α* to both H-Ras and the membrane surface in the quasi-stable state (V) slows dissociation of the transient state (III) from the membrane. Moreover, the H-Ras-bound surface state (V) is proposed to catalyze the transition to the more deeply penetrating, stable, kinase-active state bound to PIP_2_ (VI), thereby speeding the formation rate of this stable, active state. Thus, the pYp and H-Ras activators act together in synergy to create a pathway to the stable, active state with lower activation barriers and enhanced thermodynamic stability, as illustrated by comparing the synergistic path, I-II-III-V-VI, to the pYp-only path, I-II-III-IV, in Fig. 5 *B*. Notably, the net stabilization of the final H-Ras-pYp-PI3K*α*-PIP_2_ complex (path I–VI) is less than expected for simple additive stabilization by membrane and H-Ras binding (path I–IV, plus path III–V). This observation suggests that the interaction between H-Ras and PI3K*α* in the H-Ras-pYp-PI3K*α*-PIP_2_ complex perturbs the optimal PI3Kα bilayer docking geometry achieved in the pYp-PI3K*α*-PIP_2_ complex lacking H-Ras (5). Additional evidence for this perturbation is the lower specific kinase activity of the H-Ras-pYp-PI3K*α*-PIP_2_ complex relative to the pYp-PI3K*α*-PIP_2_ complex.

## Conclusions

Overall, the findings presented here reveal that simultaneous activation of PI3K*α* by a receptor activation loop and H-Ras generates strong, synergistic, activation of PI3K*α*, yielding a large increase in net kinase activity via a membrane recruitment mechanism. The findings provide important mechanistic insights into the receptor-Ras synergy that strongly activates PI3K in leukocyte chemotaxis, innate immunity, and inflammation, as well as in carcinogenesis. These insights also have implications for drug design targeting PI3K-catalyzed PIP_3_ production in carcinogensis or inflammation. The findings suggest that drugs designed to block activation of the PI3K SH2 domains by receptor phospho-Tyr residues will provide the strongest PI3K inhibition, since phospho-Tyr occupancy of the SH2 domains is required for kinase activation by receptor alone, or by synergistic receptor-Ras activation. On the other hand, the findings indicate that drugs designed to block the interaction between H-Ras and PI3K should provide nearly the same degree of PI3K inhibition, since H-Ras dominates the synergistic activation. Moreover, the finding that H-Ras binding actually inhibits, rather than activates, the PI3K*α* lipid kinase alleviates the potential concern that drug binding to the Ras binding domain might prevent Ras association but inadvertently activate PIP_3_ production via allosteric kinase regulation. Planned studies will further test the model of Fig. 5 and determine the rate constants for each step in Fig. 5 *A*. In addition, it is important to ascertain whether this simple synergy mechanism observed for the H-Ras/PI3K*α* regulatory pair is generalizable to all G protein-PI3K pairings, or whether specialized activation mechanisms exist for specific pairings of PI3K isoforms with G proteins of the Ras, Rho, and G*βγ* families.

## Author Contributions

Conception, J.J.F.; Experimental design, T.C.B., B.P.Z., and J.J.F.; Data collection, T.C.B.; Data analysis, T.C.B., B.P.Z., and J.J.F.; Data interpretation, T.C.B., B.P.Z., and J.J.F.; Other essential materials, advice, and context, R.L.W. and G.R.M.

## Supporting Material

Document S1. Figs. S1–S5 and Table S1

Document S2. Article plus Supporting Material
