# Supplementary material for: Single-Molecule Study Reveals How Receptor and Ras Synergistically Activate PI3Kα and PIP3 Signaling
Source: Biophys J. 2017 Dec 5;113(11):2396–405. doi: 10.1016/j.bpj.2017.09.018 (PMC5738497; doi:10.1016/j.bpj.2017.09.018)
Supplement: Document S1. Figs. S1–S5 and Table S1 [file mmc1.pdf]

Biophysical Journal, Volume *113*

Supplemental Information

Single-Molecule Study Reveals How Receptor and Ras Synergistically Activate PI3Kα and PIP_3 Signaling

Thomas C. Buckles, Brian P. Ziemba, Glenn R. Masson, Roger L. Williams, and Joseph J. Falke

SUPPORTING MATERIAL, SUPPLEMENTAL FIGURES S1-S5 AND TABLE S1

Mechanism by which Receptor and H-Ras Synergistically Activate PI3Kα and PIP_3 Signaling: A Single Molecule TIRFM Study

Thomas C. Buckles, Brian P. Ziemba, Glenn R. Masson, Roger L. Williams & Joseph J. Falke

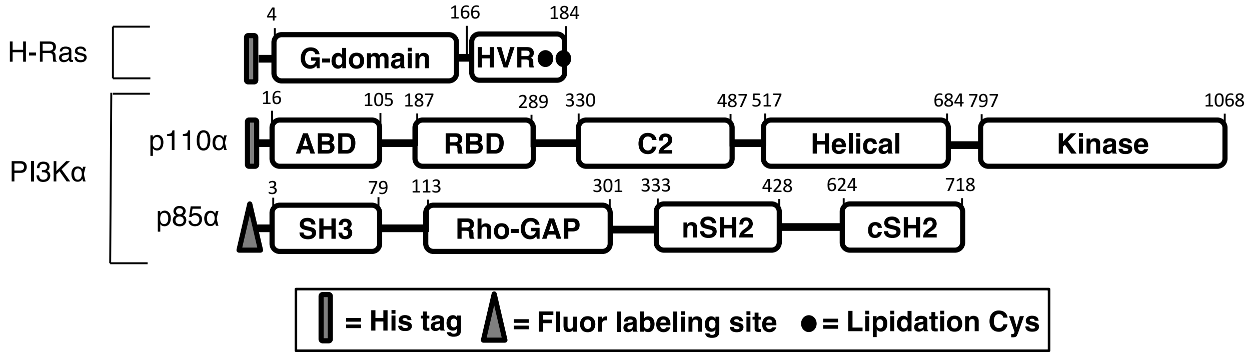

<u>Figure S1.</u> **Modular representation of the H-Ras and PI3Kα proteins used in this study.** The <u>H-Ras 1-184 construct</u> utilized was previously developed by the Groves laboratory (1) and possesses an N-terminal 6xHis affinity purification tag, and a mutation (C118S) that removes a surface-exposed Cys residue in the GTPase domain while retaining two C-terminal lipidation Cys residues (Cys 181, 184). The latter two Cys residues are palmitoylated in native H-Ras and are used for membrane anchoring in this study. Finally, the construct deletes the two, C-terminal 185 and 186 residues of native, mature H-Ras. Except where otherwise noted (Fig. S3, Table S1), H-Ras loaded with the non-hydrolyzable GTP analogue GMPPNP is employed in all experiments, both in the main text and Supplementary Materials.

The full length recombinant human <u>phosphoinositide-3-kinase alpha</u> (PI3Kα) employed was described in our previous publications (2,3) and possesses an N-terminal 6xHis affinity purification tag on its p110 catalytic subunit, as well as an N-terminal enzymatic Sfp-labeling tag on its p85 regulatory subunit.

Methods. H-Ras was recombinantly expressed and purified as described previously (4). To load with the desired nucleotide, a nucleotide replacement reaction was performed as previously detailed (1) by buffer exchanging purified H-Ras into an EDTA rich stripping buffer (150 mM NaCl, 50 mM EDTA, 1 mM TCEP, 20 mM Hepes, pH 7.4) followed by a second exchange into EDTA free, Mg^{2+} containing kinase buffer (100 mM KCl, 15 mM NaCl, 2 mM EGTA, 1.9 mM Ca^{2+}, 5 mM Mg^{2+}, 1 mM TCEP, 20 mM HEPES, pH 6.9). The nucleotide of choice was then added in excess of H-Ras followed by a 20 min incubation at 21 ºC. Finally, aliquots were snap frozen in liquid N_2 and stored at -80 ºC. When samples are thawed on ice and used in experiments, the added nucleotide is included at a concentration of 1 mM in all experimental buffers, to ensure that any nucleotide that dissociates is replaced.

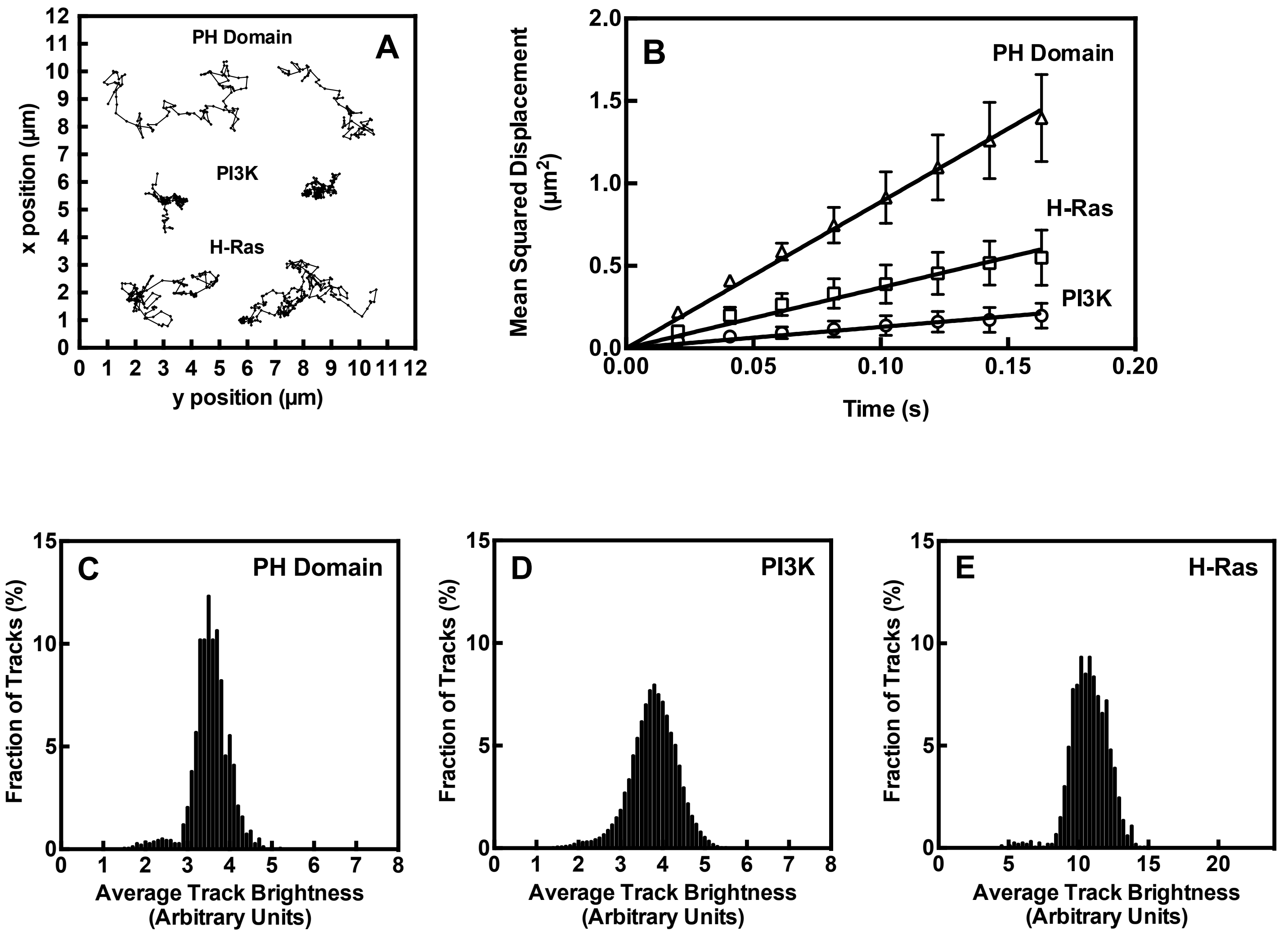

<u>Figure S2.</u> **Detecting and quantifying single protein molecules diffusing in 2-dimensions on the target supported lipid bilayer.** (**A**) Single molecule TIRFM reveals individual, fluor-tagged molecules diffusing in two dimensions on the supported bilayer surface. GRP-PH domain and PI3K are covalently tagged with Alexa Fluor 555 for visualization, while H-Ras (2-Cys construct, see Table S1) is detected by the binding of fluor-tagged anti-H-Ras antibody. (**B**) Mean square displacement (MSD) plots of the single molecule diffusion data for populations of each protein. (**C-E**) Relative brightness distributions for populations of each protein, where brightness of a protein is averaged over its track lifetime. Units are arbitrary but are normalized in the same fashion. The PH domain and PI3K each possess a single fluor coupled to their Sfp labeling tag, and are monomeric, and thus exhibit the same average brightness, within error. The 3-fold greater brightness of H-Ras is due to the presence of three fluors, on average, covalently coupled to surface Lys residues on each anti-H-Ras antibody. Thus, the observed average brightness of antibody-labeled H-Ras provides further support for a monomeric H-Ras protein under native reducing conditions, as observed previously (9).

Methods. Rabbit anti-Ras antibody (ab69747) was obtained from Abcam and labeled using excess AlexaFlour-555 succinimidyl ester via a standard procedure (5). Free fluorophore was removed by buffer exchange in Vivaspin 500 spin concentrators (30k MWCO). Unless indicated otherwise, standard supported lipid bilayers were employed as target membranes in all experiments, both in the main text and in the Supplemental Figures. These bilayers were composed of four lipids, DOPE : DOPS : $DOPIP_2$: DOPE-MCC in mole ratios 73:25:1:1. TIRFM procedures employed to track single particles, analyze 2D diffusion, and quantitate brightness distributions are described in Methods and in our previous publications (2,3,5-8). Data in B-E are averages over 4 replicates acquired on 2 or more days, 22 °C.

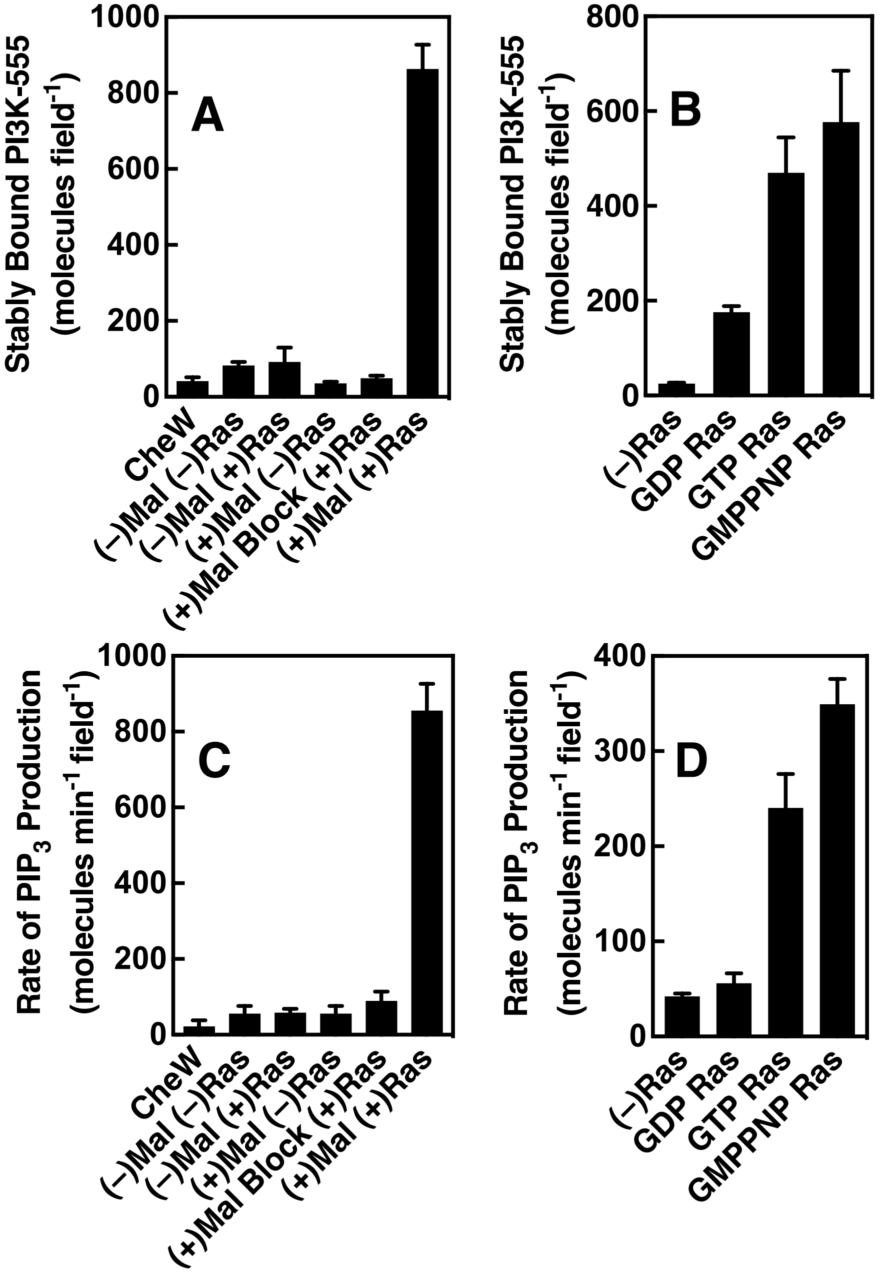

Figure S3. **Synergistic phospho-Tyr peptide and H-Ras stimulation of PI3Kα: Controls examining the H-Ras membrane anchoring and nucleotide requirements.** Each single molecule TIRFM measurement included RTK-derived phospho-Tyr peptide (pYp) to generate basal PI3Kα activation. **(A,C)** Synergistic PI3Kα activation triggered by pYp and anchored H-Ras is lost when H-Ras coupling to the supported bilayer is prevented by (i) omission of maleimide lipid (Mal), or (ii) omission of H-Ras (Ras), or (iii) glutathione quenching of the maleimide lipid prior to H-Ras addition (Mal Block). Similarly, no additional PI3Kα activation is observed when a different protein is coupled to the supported bilayer (CheW, a hydrophobic protein similar in size to H-Ras). **(B,D)** Synergistic PI3Kα activation is lost when the membrane-anchored H-Ras is occupied by GDP, while GTP and non-hydrolyzable GTP analogue GMPPNP exhibit similar synergies.

Methods. Previously described single molecule TIRFM procedures were employed, as described in Methods and in our previous publications (2,3,5-8). H-Ras was loaded with GMPPNP except where noted otherwise. The PI3Kα lipid kinase is the least stable component and exhibits some day-to-day variability, as illustrated by the variation between averages determined for data obtained on only 2 days (compare rightmost bars in C,D). For this reason, the averages in main text Figs 2-4 are for data obtained on 5+ days. Experiments at 22 ºC, averages over 9-15 replicates on ≥ 2 days, errors SEM.

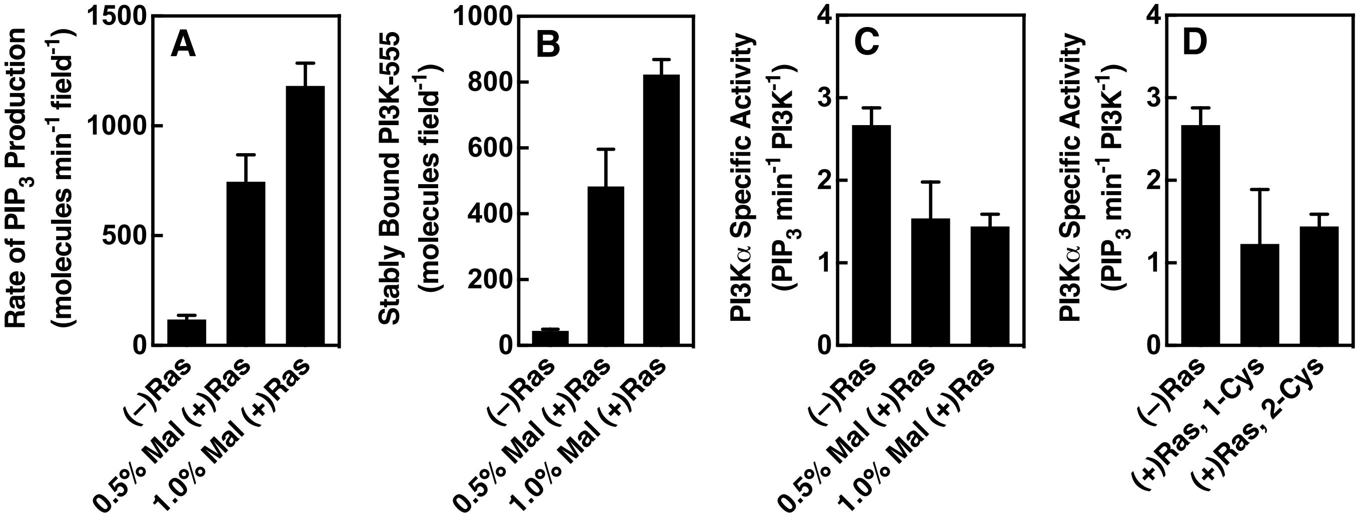

<u>Figure S4.</u> **Synergistic phospho-Tyr peptide and H-Ras stimulation of PI3Kα: Controls examining the dependence on H-Ras density and number of lipidation sites.** Each single molecule TIRFM measurement included RTK-derived phospho-Tyr peptide (pYp) to generate basal PI3Kα activation. **(A,B,C)** In all data reported in this study, H-Ras is coupled to membranes containing 1.0% maleimide lipid, except where noted as 0.5% maleimide lipid in these three panels. The H-Ras coupling to membranes containing maleimide lipid is carried out with ≥ 1000 fold mole excess H-Ras over maleimide for 5 min. When the density of maleimide lipid is reduced 2-fold, panels (A,B) show that the rate of PIP_3 production by PI3Kα and the density of stably bound PI3Kα decrease proportionally (within error). Thus, as panel (C) shows, the specific kinase activity of the average membrane-bound PI3Kα molecule is the same on both membranes (within error). These data are consistent with a simple linear dependence of the net PI3Kα membrane binding and kinase activity on the density of membrane-coupled H-Ras, while the kinase activation mechanism and specific activity in the membrane-bound state are independent of H-Ras density. **(D)** In all data reported in this study, 2-Cys H-Ras possessing two lipidation Cys residues (C181, C184) is coupled to membranes containing maleimide lipid, except where noted in panel (D) and Table S1 for 1-Cys H-Ras possessing a single lipidation Cys (C181, C184S). This panel shows that the specific kinase activity of the average membrane-bound PI3Kα molecule is the same for 2-Cys H-Ras, which is coupled predominantly by both Cys residues (see Table S1), and for 1-Cys H-Ras coupled via a single Cys.

Methods. Previously described single molecule TIRFM procedures were employed, as described in Methods and in our previous publications (2,3,5-8). Data at 22 ºC are averages over 7-9 replicates on at least 2 days. Error bars are standard errors of the mean (A,B) or propagated standard errors of the mean (B,C).

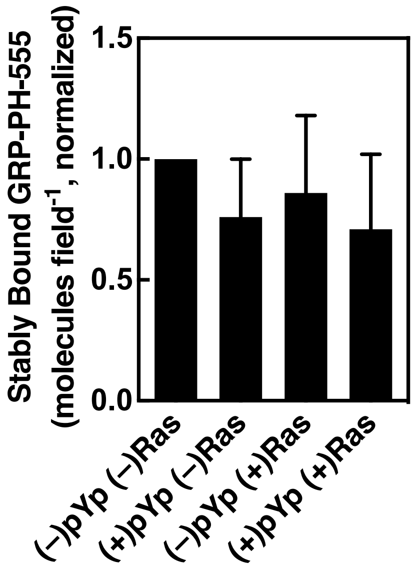

Figure S5. Controls examining the effects of PI3K activators on the binding of PH domain to PIP_3. Single molecule TIRFM was employed to quantitate the binding of the PIP_3 sensor employed in this study, Alexa Fluor 555-tagged GRP PH domain, to its target lipid. Relative to the absence of activators ((–)pYp (–)H-Ras, normalized to 1.0)), the presence of the PI3K activators RTK pYp and/or membrane-coupled H-Ras were found to have no significant effect on the density of PIP_3 sensor molecules on the supported bilayer surface. Thus, the effects of activators on PI3Kα lipid kinase activity measured in the single molecule TIRFM assay (Figs. 2 and S3) can be attributed to the effects of the activators on the enzyme, rather than on PIP_3 sensor binding to product.

Methods. Previously described single molecule TIRFM procedures were employed, as described in Methods and in our previous publications (2,3,5-8). Data are averages over 6 replicates on 2 days. Error bars are standard errors of the mean.

Protein	Density (per μM^2)	D_{Fast} (μM^2/s)	Fraction Fast	D_{Slow} (μM^2/s)	Fraction Slow
H-Ras (2 Cys) GMPPNP[a,b]	0.8 ± 0.1	1.3 ± 0.1	0.26 ± 0.01	0.7 ± 0.1	0.74 ± 0.01
H-Ras (2 Cys) GMPPNP[a]	0.5 ± 0.1	1.7 ± 0.4	0.28 ± 0.05	0.7 ± 0.1	0.72 ± 0.05
H-Ras (2 Cys) GTP[a]	0.7 ± 0.3	1.9 ± 0.5	0.31 ± 0.25	0.4 ± 0.1	0.69 ± 0.03
H-Ras (2 Cys) GDP[a]	0.8 ± 0.2	1.3 ± 0.3	0.35 ± 0.03	0.4 ± 0.3	0.65 ± 0.02
H-Ras (1 Cys)[a]	0.8 ± 0.1	1.4 ± 0.1	1	-	-
GRP-PH-555	N.D.	1.6 ± 0.2	1	-	-

[a] Detected by the binding of anti-H-Ras antibody labeled with Alexa Fluor 555
[b] Same, but half the usual saturating concentration of antibody

Table S1. Controls comparing the surface densities and diffusion characteristics of different H-Ras nucleotide binding states and different numbers of coupled lipids. H-Ras loaded with the indicated nucleotide was covalently coupled to maleimide-PE lipid on supported bilayers. Individual molecules were visualized by the binding of fluor-tagged anti-H-Ras antibody, and 2D single molecule diffusion tracks were analyzed to determine the diffusion characteristics of the single molecule population. Some variation in H-Ras surface density is observed between experiments on different days, due to variability in the efficiency of maleimide-PE into the bilayer lipid mixture, and variability in the coupling efficiency of protein reacting with the maleimide headgroup.

The 2 Cys H-Ras construct was used in all experiments in the main text and Supporting Materials, except where noted. This construct exhibits a major, slowly diffusing monomeric population coupled via both Cys 181 and 184 residues to two maleimide-PE lipids, as well as a minor, rapidly diffusing monomeric population coupled via a single Cys residue to one lipid (monomeric state defined by Fig. S2C-E). Comparison of H-Ras loaded with different nucleotides reveals no major differences between density or diffusion parameters, within the error of measurement: none of the observed differences are statistically significant. Specifically, the error bars overlap for all pairs of parameters measured for different bound nucleotides, with one exception: the error bars measured for the slow diffusion constants of H-Ras loaded with GMPPNP and GTP do not overlap, but the calculated p value (0.16) for this difference indicates it is not significant. Previous studies of 2-Cys H-Ras loaded with fluorescent GTP and GDP analogues also found that nucleotide substitution had no detectable effect on the brightness or diffusion of H-Ras monomers when light-induced dimerization was suppressed by the presence of reducing agent (9). All of our studies include a physiological level of the cytoplasmic reducing agent glutathione (5 mM); thus, as predicted (9), light-induced dimerization is not observed and H-Ras is monomeric.

The 1 Cys H-Ras construct possesses the C184S mutation, and thus retains only one Cys residue for membrane coupling. This 1 Cys construct exhibits a homogeneous diffusion within error of that observed for the fast component of the 2 Cys construct diffusion, confirming that both fast diffusers are monomeric H-Ras coupled to a single lipid. Moreover, both fast diffusers exhibit diffusion constants indistinguishable from that of PH domain bound to a single PIP_3; thus, as expected, monomeric proteins each bound to a single DO lipid exhibit the same diffusion speed defined by the frictional drag of the bound lipid (5).

Methods. Saturating (40 nM) antibody was added to supported bilayers decorated with H-Ras coupled to maleimide-PE, using a 1:30 ratio of fluor-labeled to dark H-Ras to ensure countable densities of fluorescent H-Ras molecules. The same TIRFM procedures described in Methods and Figs. S2-S4 were employed, except that PH domain was imaged on supported bilayers containing 1 mole percent PIP_3 rather than PIP_2. Data are averages of 6+ replicates acquired on 2+ days, 22 °C.

References for Supporting Material

1. Lin W.C., L. Iversen, H.L. Tu, C. Rhodes, S.M. Christensen, J.S. Iwig, S.D. Hansen, W.Y. Huang, and J.T. Groves (2014) *H-Ras forms dimers on membrane surfaces via a protein-protein interface.* Proc Natl Acad Sci U S A **111**(8):2996-3001.
2. Ziemba, B. P., J. E. Burke, G. Masson, R. L. Williams, and J. J. Falke (2016) *Regulation of PI3K by PKC and MARCKS: Single-Molecule Analysis of a Reconstituted Signaling Pathway*. Biophys J. **110**:1811-1825.
3. Ziemba B.P., G.H. Swisher, G. Masson, J.E. Burke, R.L. Williams, and J.J. Falke (2016) *Regulation of a Coupled MARCKS-PI3K Lipid Kinase Circuit by Calmodulin: Single-Molecule Analysis of a Membrane-Bound Signaling Module.* Biochemistry **55**(46):6395-6405.
4. Gureasko J., W.J. Galush, S. Boykevisch, H. Sondermann, D. Bar-Sagi, J.T. Groves, and J. Kuriyan (2008) *Membrane-dependent signal integration by the Ras activator Son of sevenless* Nat Struct Mol Biol **15**(5):452-61.
5. Ziemba, B.P. and J.J. Falke (2013) *Lateral diffusion of peripheral membrane proteins on supported lipid bilayers is controlled by the additive frictional drags of (1) bound lipids and (2) protein domains penetrating into the bilayer hydrocarbon core.* Chemistry and Physics of Lipids **172-173**:67-77.
6. Knight J.D. and J.J. Falke (2009) *Single-molecule fluorescence studies of a PH domain: new insights into the membrane docking reaction*. Biophys J. **96**(2):566-82.
7. Knight J.D., M.G. Lerner, J.G. Marcano-Velázquez, R.W. Pastor and J.J. Falke (2010) *Single molecule diffusion of membrane-bound proteins: window into lipid contacts and bilayer dynamics.* Biophys J. **99**(9):2879-87.
8. Ziemba, B.P., C. Pilling, V. Calleja, B. Larijani, and J.J. Falke (2013) *The PH domain of phosphoinositide-dependent kinase-1 exhibits a novel, phospho-regulated monomer-dimer equilibrium with important implications for kinase domain activation: single-molecule and ensemble studies.* Biochemistry **52**(28):4820-9.
9. Chung, J.K., Y.K. Lee, H.Y. Lam, and J.T. Groves (2016) *Covalent Ras Dimerization on Membrane Surfaces through Photosensitized Oxidation.* J Am Chem Soc. **138**:1800-3.
